# Thalamocortical circuits for the formation of hierarchical pathways in the mammalian visual cortex

**DOI:** 10.3389/fncir.2023.1155195

**Published:** 2023-04-17

**Authors:** Tomonari Murakami, Kenichi Ohki

**Affiliations:** ^1^Department of Physiology, Graduate School of Medicine, The University of Tokyo, Tokyo, Japan; ^2^Institute for AI and Beyond, The University of Tokyo, Tokyo, Japan; ^3^World Premier International Research Center Initiative-International Research Center for Neurointelligence (WPI-IRCN), The University of Tokyo, Tokyo, Japan

**Keywords:** development, neural circuits, visual system, visual cortex, thalamocortical projections, spontaneous activity, mouse

## Abstract

External sensory inputs propagate from lower-order to higher-order brain areas, and the hierarchical neural network supporting this information flow is a fundamental structure of the mammalian brain. In the visual system, multiple hierarchical pathways process different features of the visual information in parallel. The brain can form this hierarchical structure during development with few individual differences. A complete understanding of this formation mechanism is one of the major goals of neuroscience. For this purpose, it is necessary to clarify the anatomical formation process of connections between individual brain regions and to elucidate the molecular and activity-dependent mechanisms that instruct these connections in each areal pair. Over the years, researchers have unveiled developmental mechanisms of the lower-order pathway from the retina to the primary visual cortex. The anatomical formation of the entire visual network from the retina to the higher visual cortex has recently been clarified, and higher-order thalamic nuclei are gaining attention as key players in this process. In this review, we summarize the network formation process in the mouse visual system, focusing on projections from the thalamic nuclei to the primary and higher visual cortices, which are formed during the early stages of development. Then, we discuss how spontaneous retinal activity that propagates through thalamocortical pathways is essential for the formation of corticocortical connections. Finally, we discuss the possible role of higher-order thalamocortical projections as template structures in the functional maturation of visual pathways that process different visual features in parallel.

## Introduction

The human cerebral cortex is divided into more than 100 areas processing various sensory inputs from the outside world (Economo and Koskinas, 1925; Triarhou, 2007; Glasser et al., 2016; Van Essen and Glasser, 2018; Van Essen et al., 2019). The visual cortex is the most developed sensory area in primates, with more than 30 cortical areas (Ungerleider and Mishkin, 1982; Zeki and Shipp, 1988; Felleman and Van Essen, 1991; Goodale and Milner, 1992; Orban et al., 2004; Markov et al., 2014). External visual inputs received by the retina propagate to the primary visual area (V1) *via* the dorsal lateral geniculate nucleus (dLGN) in the thalamus. Then, V1 sends visual information to the secondary visual cortex (V2). V2 sends projections to numerous higher-order cortical visual areas (HVAs) which are hierarchically connected (Felleman and Van Essen, 1991). The representation of information in individual cortical areas becomes more complex along this hierarchical pathway. In addition to the hierarchy, the interareal connections constitute dorsal and ventral pathways rather than a single pathway (Felleman and Van Essen, 1991; Nassi and Callaway, 2009; Kravitz et al., 2011, 2013). Visual information regarding object motion (low spatial resolution and high speed) is processed in the dorsal pathway (Kravitz et al., 2013), whereas information regarding object recognition (high spatial resolution and no motion) is processed in the ventral pathway (Kravitz et al., 2011). Furthermore, V1 sends projections to a higher-order thalamic nucleus, the pulvinar, which has bidirectional connections with HVAs and different subregions within the pulvinar are connected to the ventral and dorsal pathways (Shipp, 2003; Kaas and Lyon, 2007; Sherman, 2016; Kaas and Baldwin, 2019). Although the function of the pulvinar remains unclear, it works in gating visual information processing in V1 (Purushothaman et al., 2012), and when V1 is damaged projections from the pulvinar to the dorsal stream likely contribute to the preservation of some visual functions (blindsight; Kaas and Baldwin, 2019).

**FIGURE 1:**
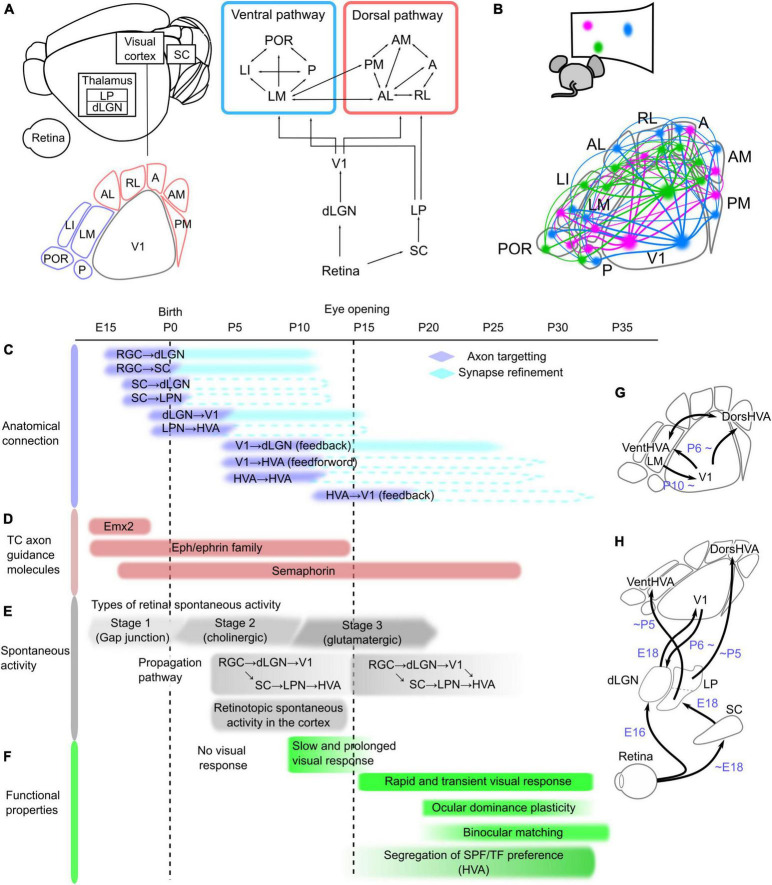
Network structure and developmental timeline of the mouse visual system. **(A)** Schematic of the neural network in the mouse visual system, which is composed of the retina, dorsal lateral geniculate thalamic nucleus (dLGN), superior colliculus (SC), lateral posterior thalamic nucleus (LPN), and visual cortex, including the primary (V1) and higher-order visual areas (HVAs). These areas are connected hierarchically. The retina sends projections to the dLGN and SC, and these regions send projections to V1 and the LPN, respectively. At least nine HVAs surround V1 in the mouse brain. HVAs receive visual information through the dLGN–V1 or SC–LPN pathway. HVAs are categorized into dorsal and ventral pathways, and the connections between HVAs belonging to the same pathway are stronger than those across pathways. **(B)** Corticocortical connections among V1 and HVAs maintain the information of object positions in the visual field, which is retinotopy. The colors in the bottom figure correspond to the locations in the visual field shown above, and the same color regions of V1 and HVAs are connected. Developmental timeline of the mouse visual system in terms of anatomical connections **(C)**, expression of thalamocortical (TC) axon guidance molecules **(D)**, spontaneous activity **(E)**, and functional properties of neurons in V1 and HVAs **(F)**. The right schematics **(G,H)** illustrate the timing of axon targeting for each areal pair. **(C)** Axonal projections reach the target area and undergo refinement to the synapse in the precise area. The timing of the formation of axon projections from the retina to V1 and of corticocortical connections between V1 and HVAs has been elucidated, and the process of synapse refinement has also been demonstrated in the pathways from the retina to the dLGN and SC, and from the dLGN to V1 (shown as filled boxes). However, it is not yet known whether the pathways from the SC to the dLGN or LPN, from the LPN to HVAs, and the connections between V1 and HVAs undergo synapse refinement (indicated by dashed lines). **(D)** Timing of expression of representative axon guidance molecule families that form thalamocortical projections from the dLGN to V1. Eph/ephrin and semaphorin families have many subtypes that are expressed at different developmental stages, and they are all shown together in this figure. **(E)** Retinal activity during development is classified into three types that are mediated by different forms of neurotransmission. The first stage is mediated by a combination of gap junctions and cholinergic circuits (–P0), the second by cholinergic activity (P0–P10), and the third by glutamatergic activity (P10–). The propagation of these spontaneous retinal activities to the cortex is confirmed at P5, however, because V1-HVA projections are not fully formed at this time, retinal activity propagates to V1 and HVA *via* different pathways. After the formation of corticocortical projections, spontaneous activity propagates not only from the thalamus but also *via* V1-HVA projections. **(F)** Slow and prolonged visual responses were observed around P10. After eye opening, the visual cortex obtains rapid visual response with the short decay time. At this time, thalamocortical and corticocortical projections are still developing, and functional maturation of neurons, such as ocular dominance plasticity and binocular matching of orientation selectivity, starts approximately 1 week after eye opening. Furthermore, differences in spatial and temporal frequencies (SPF and TF) preference among HVAs became more pronounced just after eye opening to approximately 2 weeks later. **(G,H)** Simple schematics showing axonal projections reaching the target area in the visual pathways between V1 and HVAs **(G)** and from the retina to V1 and HVAs **(H)**.

The hierarchical dorsal/ventral pathways and thalamic–cortical loops in primates are also present in the mouse visual system (Figure 1A; Wang et al., 2012; Glickfeld et al., 2014; Zingg et al., 2014; Seabrook et al., 2017; Bennett et al., 2019; Harris et al., 2019; Siegle et al., 2021; D’Souza et al., 2022). The topographic information in visual inputs, retinotopy, is retained throughout visual pathways from the retina to V1 and HVAs (Glickfeld et al., 2014; Seabrook et al., 2017). Burkhalter’s group analyzed the retinotopic projection patterns from V1 to HVAs and found that there are at least 10 HVAs with retinotopic structures around V1 (Wang and Burkhalter, 2007). They further examined the connectivity patterns between all cortical areas and claimed that HVAs are divided into ventral and dorsal pathways (Wang et al., 2011, 2012). Subsequently, several studies have functionally demonstrated that each HVA processes different visual information in parallel (Andermann et al., 2011; Marshel et al., 2011; Murakami et al., 2017). Recently, the Allen Institute and Burkhalter’s groups have proposed an anatomical and electrophysiological hierarchy among HVAs (Harris et al., 2019; Siegle et al., 2021; D’Souza et al., 2022). Moreover, the lateral posterior nucleus (LPN), which is thought to correspond to the pulvinar in the primate visual system (Zhou et al., 2017), has reciprocal connections with all HVAs, and the subregions of the LPN have connections with HVAs in the ventral and dorsal pathways (Tohmi et al., 2014; Bennett et al., 2019; Juavinett et al., 2019). Thus, the mouse visual system is similar to that of primates, although there are some differences such as a number of cortical areas and subregions of thalamic nuclei in the visual system. Furthermore, due to the recent development of genetic methods and convenience of experiments, mice are widely used as model animals for studying visual information processing.

One of the key questions in neuroscience is how the network structure of the visual system common to primates and rodents is formed during development. There are two functional goals to be achieved in the formation of neural networks: (1) to form interareal connections that preserve the retinotopic structure, and (2) to establish neural pathways that allow HVAs to process different features in the visual information in parallel. A single brain area contains several million neurons that project to precise locations that reflect the retinotopic structure of the target area (Figure 1B). Furthermore, they are wired to propagate different visual information when projecting from one area to multiple target areas (Glickfeld et al., 2013; Matsui and Ohki, 2013; Han et al., 2018; Blot et al., 2021). What strategies do our brains employ to achieve their functional goals? To determine this, we need to know the anatomical formation process of interareal connections. The first step in connectivity formation is the arrival of axon projections from the source to the target region (Stoeckli, 2018). Neuronal axons reach the target brain region under the control of axon guidance molecules (Feldheim and O’Leary, 2010). At this stage, axonal projections are still roughly formed in the target area. Axon pruning and synapse formation at precise terminal positions subsequently occur with spontaneous activity (Huberman et al., 2008). Thus, the formation of connections from the retina to V1 consists of two steps and the formation timing differs between each areal pair. However, the developmental mechanisms of corticocortical connections are still unknown. Identification of the timeline of connectivity formation in the visual pathways, as well as the involvement of molecular expression or spontaneous activity patterns at that time, will provide a complete understanding of the neural network formation.

Here, we summarize the anatomical formation of neural pathways from the retina to HVAs in the mouse visual system. We then focus on recent findings that the thalamocortical pathways of dLGN–V1 and LPN–HVA are formed earlier than the connections between cortical areas, and discuss their role in the development of cortical pathways composed of V1 and HVAs. Finally, we discuss how these findings in the mouse visual system relate to the formation of neural networks in primate brains by comparing them with the results of previous studies.

## Anatomical and functional development of the mouse visual pathway

There is no direct projection from the retina to the cortex, and neural activity propagates from the retina to the cortex *via* the dLGN, superior colliculus (SC), and LPN. First, we summarize the timing of the formation of connections between these regions during development, focusing on not only the anatomically observed arrival of axonal projections to target areas but also the actual propagation of activity through these connections (Figures 1C–H). We also discuss the development of visual functions that have been observed to coincide with projection maturation.

Retinal ganglion cells project to the dLGN and SC in adults. These axonal projections arrive at the target areas around embryonic day (E) 16 (Figure 1C; Godement et al., 1984; McLaughlin et al., 2003; Herrera et al., 2019). The initial projections first elongate axons to a rough location in the target regions. Retinal waves, a pattern of spontaneous retinal activity, are observed in the dLGN and SC around postnatal day (P) 5 (Figure 1E; Murakami et al., 2022), indicating that retinal projections to the dLGN and SC are formed enough to propagate neural activity in the first few days after birth. The initial projections are followed by refinements such as axonal pruning and synapse formation in the target area (Huberman et al., 2008; Hong and Chen, 2011). This refinement process continues until around P10 when synapses are formed in precise terminal locations that reflect the retinotopic structure and ocular dominance in the dLGN and SC (Huberman et al., 2008).

Neurons in the SC send projections to the thalamic nuclei. SC axons have already innervated the dLGN and LPN prior to birth (Figure 1C; Guillamón-Vivancos et al., 2022). A pathway from the SC to V1 *via* the thalamic nucleus was reported to propagate activity upon whisker stimulation at E18 (Guillamón-Vivancos et al., 2022), suggesting that the projections from the SC to the dLGN are established enough to propagate neural activity at E18, however, whether retinal activity propagates to the SC at E18 has not been directly investigated. Regarding the propagation of retinal activity, we recently revealed that SC is a relay point to the LPN and HVAs by P5 (Murakami et al., 2022).

The next step is the formation of projections from the thalamic nuclei to the visual cortex. Neurons in the dLGN and LPN extend their axons to V1 and HVAs, respectively (Bennett et al., 2019; Murakami et al., 2022). By E18, the projections from the dLGN to V1 first arrive at the subplate under the cortical layers (Auladell et al., 2000; López-Bendito and Molnár, 2003; Price et al., 2006; Kanold, 2009). At this time, the neural response to whisker stimulation at E18 is propagated to V1 *via* the dLGN (Guillamón-Vivancos et al., 2022), indicating that thalamocortical axons arriving at the subplate are capable of propagating neural activity. The cortical layers are not yet fully established at birth, and thalamocortical axons invade the cortex in correspondence with cortical layer maturation a few days after birth (López-Bendito and Molnár, 2003). It has been demonstrated that at P5, the dLGN–V1 pathway propagates spontaneous retinal activity (Figure 1E; Murakami et al., 2022), although it takes approximately 10 days after birth to complete accurate projections to the target layer (López-Bendito and Molnár, 2003). Similarly, projections from the LPN reach HVAs at least by P5, because we have shown retinal activity propagation along this pathway at P5, similar to dLGN–V1 (Murakami et al., 2022). Conversely, this study also demonstrated that, at this age, anatomical connections from V1 to HVA (V1–HVA) and from HVA to HVA (HVA–HVA) were very few and neural activity does not propagate from V1 to HVAs. These findings indicate that the dLGN–V1 and LPN–HVA pathways are formed at approximately the same developmental stage before the corticocortical connections among V1 and HVAs. Importantly, the spontaneous activity of V1 and HVAs at P5 showed an activity pattern that reflected the retinotopic structure (Murakami et al., 2022). This indicates that the retinotopic structure in HVAs is acquired prior to the formation of corticocortical connections.

Neural connections between the thalamic nuclei and visual cortex are reciprocal (Shepherd and Yamawaki, 2021). In adults, neurons in V1 project to the dLGN, LPN, and thalamic reticular nucleus (TRN), which is composed of inhibitory neurons (Pinault, 2004). During development, feedback projections from neurons in V1 layer 6 to the dLGN are gradually formed from P6 (Grant et al., 2016). Although dLGN–V1 projections are sparse at this age, experiments using optogenetic stimulation of V1 neurons have shown that neural activity propagates from V1 to the dLGN from P6 (Murata and Colonnese, 2016). Anatomical projections from V1 to the LPN were observed at P5 (Murakami et al., 2022), although it has not yet been confirmed whether neural activity propagates along V1–LPN projections at P5. Projections from V1 to TRN emerge after the V1–dLGN projections and gradually mature until P13 (Murata and Colonnese, 2016).

Finally, corticocortical connections are formed from V1 to HVA or from HVA to HVA. Both V1–HVA and HVA–HVA projections are gradually observed from P6 and become strong enough to propagate spontaneous activity around P10 (Dong et al., 2004; Berezovskii et al., 2011; Murakami et al., 2022). These findings suggest that both V1–HVA and HVA–HVA corticocortical connections form at almost the same time. It should be noted that only one pair of HVA–HVA connections between the lateromedial (LM) and anteromedial areas is already formed at P5 (Murakami et al., 2022), which suggests the possibility that some HVA–HVA connections may also have formed earlier. In addition, corticocortical connections are bidirectional and have feedforward and feedback projections. Projections from V1 to HVAs are feedforward, and those from HVAs to V1 are feedback (Resulaj, 2021). Feedback projections from LM to V1 are gradually observed after P10 (Dong et al., 2004; Berezovskii et al., 2011), suggesting that feedback projections are formed later than feedforward projections. It remains unclear which direction is feedforward or feedback in HVA–HVA connections, because the hierarchical relationship among HVAs has not yet been causally elucidated. Both feedforward and feedback projections mature by 2–3 weeks after eye opening (Dong et al., 2004), which likely corresponds to the functional maturation of V1 and HVAs (Figure 1F).

We have summarized the process of formation of the visual neural pathway from the retina to HVAs. Although the precise timeline of the formation process in several areal pairs has not yet been confirmed, over a long period, all projections begin with a rough extension to the target area, followed by refinement, such as axon pruning and synapse formation. In the next section, we discuss the developmental mechanisms underlying the formation of interareal connections, focusing on thalamocortical projections.

## Developmental mechanisms of the thalamocortical projection from dLGN to V1

Molecular expression and spontaneous retinal activity control axon targeting and refinement, respectively. This mechanism has been well investigated in lower-order pathways, such as retina–dLGN–V1 and retina–SC. Here, we briefly describe the molecular and activity-dependent mechanisms involved in the formation of neural projections from the dLGN to V1 (Feldheim and O’Leary, 2010; Molnár et al., 2012).

Areal differentiation of the visual cortex is controlled by molecules such as Emx2, Pax6, and Fgf8 (Figure 1D; O’Leary et al., 2007; Cadwell et al., 2019). Emx2 has an expression gradient that decreases from caudal to rostral (Simeone et al., 1992), whereas Pax6 has an inverse gradient expression pattern (Muzio et al., 2002). These molecules are expressed only for a short period before birth (O’Leary et al., 2007). Knockout of Emx2 results in an extremely small visual cortex, whereas knockout of Pax6 results in a larger visual cortex and other smaller regions (Bishop et al., 2000; Hamasaki et al., 2004). Emx2 regulates Fgf8 signaling and patterns in cortical regions (Shimogori and Grove, 2003). Thus, these molecules control the initial arealization of cortical brain regions (proto map).

Next, under the control of axon guidance molecules such as Eph/ephrin, axons of dLGN neurons elongate to V1 and reach the subplate around birth (Feldheim and O’Leary, 2010). Eph/ephrin resides on the cell membrane; Eph is the receptor, and ephrin is the ligand (Drescher, 1997). These molecules have an inverse gradient expression pattern in not only dLGN and V1 but also the retina and SC, and this expression gradient allows axon extension to target areas while preserving topographic structure (Cang et al., 2005a). Knockout of these molecules disrupts the retinotopy of dLGN, SC, and V1 in adults. This strategy of determining the projection site by the molecular expression pattern of Eph/ephrin is based on the “chemical affinity theory” proposed by Sperry more than 50 years ago, which states that the projection site of axons is determined by the keyhole relationship between the molecular labels on the axon and the target cell sides. Eph/ephrin expression is high from the prenatal period to 2 weeks after birth, before the eyes are open (Dye et al., 2011a,b). Other axon guidance molecules such as semaphorin and Lhx2, are also expressed during this period (Allen Brain Atlas^1^), and abnormal projection formation from the dLGN to V1 was observed when these molecules were knocked out (Diao et al., 2018).

Axons extending into the target area by molecular control must synapse at their final position within the target area (axon refinement). Spontaneous activity generated in the retina plays an important role in this process (Huberman et al., 2008). Spontaneous retinal activity corresponding to axon refinement in projections from the retina to the dLGN and SC is acetylcholine-dependent at stage 2 because this refinement occurs from P1 to P10, before eye opening (Figure 1E; Huberman et al., 2008). Spontaneous activity during this period has a wave-like pattern that flows in the entire V1 (retinal waves), and when this pattern is disrupted using pharmacological methods or transgenic mice, the eye-specific projection patterns in the dLGN and SC are disrupted (Penn et al., 1998; Rossi et al., 2021). Similar results have been anatomically observed in retinal projections from the dLGN to V1: the retinotopic structure of V1 in adults is partially disrupted by a disruption of the activity patterns of stage 2 retinal waves (Cang et al., 2005b). Thus, the stage 2 retinal wave refines the projection from the dLGN to V1, and finally synapses at the correct location for retinotopy. In addition to the retinal activity, thalamic activity is likely involved in the axon refinement. Impairment of spontaneous activity in the ventral posteromedial nucleus (VPM) results in the disruption of barrel structures (Antón-Bolaños et al., 2019). These results raise the possibility that spontaneous retinal and thalamic activity may also play a role in axon refinement during the formation of corticocortical connections between V1 and HVAs.

Thus, the developmental mechanisms of projections from the dLGN to V1 have been investigated in detail, including molecular-controlled axon targeting and activity-dependent axon refinement. After the eyes open and retinal waves disappear, synapse refinement continues in a visual experience-dependent manner, causing functional maturation, such as ocular dominance plasticity and binocular matching of orientation selectivity in V1 neurons, until P35 (Figure 1F).

## The role of LPN–HVA pathway in the development of visual neural network

Our recent study revealed the formation process of neural network containing HVAs and LPN in the mouse visual system (Murakami et al., 2022). The LPN–HVA pathway was formed in parallel with dLGN–V1 by P5, earlier than the formation of corticocortical connections. Subsequently, corticocortical connections among V1 and HVAs are formed and complete the hierarchical network. Notably, early in development, all HVAs received projections from the LPN, which were formed independently of the dLGN–V1 pathway. Here, we discuss the functional role of thalamocortical circuits that are formed early in development.

Previous studies have shown that thalamocortical pathways from lower-order thalamic nuclei to the primary sensory cortex are involved in regional differentiation from the telencephalon during development. In the visual system, when the dLGN is genetically shrunk from early development before birth in transgenic mice, cortical areas labeled with neuronal marker genes for V1 are no longer observed (Chou et al., 2013; Vue et al., 2013). In the somatosensory cortex, genetic removal of the VPM, which normally projects to the primary somatosensory cortex (S1), results in loss of the S1 barrel structure (Pouchelon et al., 2014). These findings indicate that the early formation of thalamocortical projections is essential for the differentiation of cortical sensory areas. Interestingly, genetic shrinkage of the LPN reduces the areal size of HVAs (Vue et al., 2013). These findings indicate that thalamocortical projections can induce regional cortical differentiation.

Spontaneous activity generated in the retina propagates along thalamocortical pathways, *via* the dLGN and LPN to V1 and HVAs in parallel (Murakami et al., 2022). Spatiotemporal patterns of spontaneous activity in the visual cortex, which were visualized using functional correlation analysis, revealed retinotopy-like structures in V1 and seven HVAs at P5 (Murakami et al., 2022). These retinotopic structures reflect correlated spontaneous activity in each area, and the drastic decrease in cortical spontaneous activity after binocular enucleation at P5 indicates that correlated spontaneous activity in V1 and HVAs is derived from spontaneous retinal activity (Murakami et al., 2022). Chronic removal of retinal activity by binocular enucleation at birth results in diffusive projections from V1 to HVAs and disruption of the retinotopic structure (Murakami et al., 2022). Furthermore, the axon density of V1–HVA projections in binocular enucleated mice seemed lower than that of control mice, suggesting that the number of axonal projections decreased. Although this impression could not be directly proven because it was impossible to completely control the expression level of fluorescent proteins in each experiment, spontaneous retinal activity may be important for both axon refinement and projection targeting. Thus, our study showed that V1 and HVAs receive retinotopic information by retinal activity propagation, and suggested the possibility that synchronous spontaneous activity in retinotopically corresponding regions in V1 and HVAs affects the formation of corticocortical connections between V1 and HVAs. The Hebbian rule suggests that neural connections between neurons with synchronized neural activity are enhanced, while other connections are attenuated. Therefore, synchronous activity occurring in retinotopically corresponding regions in V1 and HVAs may be the basis for the formation of retinotopic connections among V1 and HVAs according to the Hebbian rule.

**FIGURE 2:**
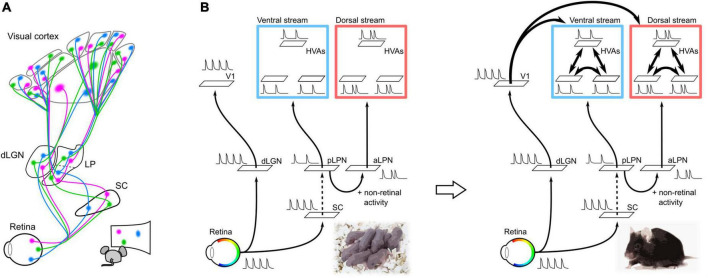
Neural pathways and spontaneous retinal activity propagation in the developing mouse brain. **(A)** Schematic of the visual neural network at P5. V1 and HVAs receive retinal activity along parallel pathways from the retina *via* the dLGN or LPN in the developing mouse brain at P5 (parallel module structure). Spontaneous retinal activity contains retinotopic information, which allows all visual areas to obtain retinotopic maps before the formation of corticocortical connections. **(B)** Hypothesis of the propagation of retinal activity at P5. The spontaneous activities of V1 and HVA at P5 were not perfectly synchronized, even though they were of retinal origin. This suggests that spontaneous retinal activity underwent some decorrelation before propagating to the cortex. The retina–dLGN–V1, retina–SC, and LPN-HVA pathways likely propagate spontaneous activity with high transfer efficiency. Conversely, projections from the SC to the LPN may have a low transfer efficiency and cause a drop in retinal activity. In addition to the transfer efficiency, there is a difference in spontaneous activity between HVAs in the dorsal and ventral pathways, which may be due to the addition of non-retinal derived activity in the aLPN.

It is possible that altered thalamic and cortical activity patterns and molecular expression may underlie the disruption of thalamocortical projections due to the chronic removal of retinal activity. The lack of retinal activity replaces spontaneous activity in the visual cortex from a local spot-like activity pattern to a broader activity pattern (Murakami et al., 2022). This broader activity pattern may prevent refinement of axon projections, resulting in diffuse cortical projections. Furthermore, the chronic removal of retinal activity alters the expression patterns of axon guidance molecules and other molecules in the cortex before cortical connections are formed (Dye et al., 2012; Pouchelon et al., 2014). Changes in gene expression patterns upon chronic removal of retinal activity have also been observed in the thalamic nuclei of the LGN and LPN (Frangeul et al., 2016). Thus, failure of retinal activity propagation to the thalamic and cortical regions likely results in adaptive changes in spontaneous activity and gene expression patterns, thereby impairing cortical connectivity. In other words, parallel thalamocortical pathways (dLGN–V1 and LPN–HVAs) propagate retinotopic information to V1 and HVA to guide the formation of corticocortical connections (Figure 2A).

## Possible roles of LPN–HVA pathway in the functional segregation and differentiation of dorsal and ventral pathways

A second role of thalamocortical pathways from the LPN to HVAs may be to induce specific areal connections between HVAs within the ventral or dorsal pathways. In adults, different subregions within the LPN send projections to HVAs in the ventral and dorsal pathways (Bennett et al., 2019). HVAs in the ventral pathway receive axonal projections from the posterior part of LPN (pLPN) whereas HVAs in the dorsal pathway receive projections from the anterior part of LPN (aLPN). The parallel module structures of the pLPN–ventral HVA (vHVA) and aLPN–dorsal HVA (dHVA) are formed by P5. Reflecting this structure, spontaneous activity between vHVAs or between dHVAs is more highly correlated than that between vHVAs and dHVAs (Murakami et al., 2022). The decorrelation of spontaneous activity between vHVAs and dHVAs is likely due to the difference in activity propagation from the LPN to HVAs, as we discuss in the next section. We believe that the decorrelation of spontaneous activity between vHVAs and dHVAs results in activity synchronization within the same pathway. Synchronization of spontaneous activity within the same pathway may strengthen connections between HVAs within the same pathway according to the Hebbian rule (Figure 2B; Butts et al., 2007; Lee et al., 2014; Leighton and Lohmann, 2016).

A third role of the LPN–HVA pathways may be to act as templates for the functional differentiation of cortical ventral and dorsal pathways. In adults, HVAs in the ventral and dorsal pathways process different visual information. For example, vHVAs prefer visual inputs with high spatial frequency (SPF) and low temporal frequency (TF) whereas dHVAs prefer those with low SPF and high TF (Andermann et al., 2011; Marshel et al., 2011; Murakami et al., 2017). The distribution of different visual information from V1 to HVAs is thought to be important for the functional differentiation among HVAs (Glickfeld et al., 2013; Matsui and Ohki, 2013). Therefore, the formation of corticocortical connections between V1 and HVAs likely requires axon refinement to acquire not only retinotopic structures but also functional differentiation in HVAs. The projections from V1 to HVAs begin to propagate spontaneous activity strongly from around P10 and their axon density gradually increases until a few weeks after eye opening (Dong et al., 2004; Berezovskii et al., 2011). In contrast, the LPN–HVA connectivity pattern is maintained from a few days after birth to adulthood, and propagates retinal activity in neonates and visual information in adults. The strong propagation of spontaneous activity along the retina–SC–LPN–HVA pathway a few days after birth suggests that this pathway is well established before eye opening. Therefore, visual information is expected to propagate along this pathway when the visual experience begins with the eye-opening. This visual information propagating to HVAs *via* the LPN may be involved in the differences in spatiotemporal preference among HVAs at eye opening (Murakami et al., 2017). What kind of visual information is sent from V1 and LPN to HVAs at eye opening is an interesting question. If the LPN–HVA pathways at eye opening convey different visual information to each HVA, projections from V1 to HVAs may be refined using the visual inputs from LPN–HVA pathways as the instruction signal. This case raises the possibility that LPN–HVA pathways serve as a template structure for the acquisition of the distribution of visual information from V1 to HVAs.

## Decorrelation of retina-derived spontaneous activity in V1 and dorsal/ventral pathways

The correlation of spontaneous activity between HVAs is higher than that between V1 and HVA before eye opening (Murakami et al., 2022). Furthermore, spontaneous activity between vHVAs or between dHVAs is more highly correlated than that between vHVAs and dHVAs at this developmental stage. Because spontaneous activity in V1 and HVAs is mainly derived from the retina, if retinal activity fully propagates to the visual cortex *via* parallel modules, V1 and HVAs have high correlated activity, but actually not. What decorrelates the spontaneous activity between V1, vHVAs, and dHVAs?

Spontaneous activity in vHVAs almost disappeared after binocular enucleation at P5, whereas that in dHVAs remained. This result suggests that spontaneous activity in vHVAs is derived from the retina, whereas dHVAs have both retinal and non-retinal activities (Murakami et al., 2022). As pharmacological inactivation of the LPN greatly reduces the spontaneous activity of dHVAs, the non-retinal activity of dHVAs likely propagates from the aLPN (Murakami et al., 2022). It is expected that the non-retinal activity in the aLPN likely decorrelates with spontaneous activity between the pLPN and aLPN, and further between the ventral and dorsal pathways.

In addition to non-retinal activity, the transfer efficiency of retinal activity propagation along the dLGN–V1 and LPN–HVA pathways likely contributes to the decorrelation of spontaneous activity between V1 and HVAs. If retinal activity is fully transmitted to the subcortical and cortical regions, spontaneous activity in V1 and vHVAs will be highly correlated, however, this was not the case. This result implies that the activity decorrelation between V1 and vHVAs may be due to differences in the transfer reliability of retinal activity between pathways from the retina to V1 or vHVAs. A previous study reported high transfer efficiency from the retina to the SC and V1 (Ackman et al., 2012). In addition, we have revealed high transfer efficiency in dLGN–V1 and pLPN–vHVA pathways (Murakami et al., 2022). These results imply that projections from the SC to pLPN may have low transfer efficiency, resulting in activity decorrelation between the dLGN and pLPN. Indeed, spontaneous activities of the dLGN and pLPN were not completely synchronized, and some activities were absent in the pLPN (Murakami et al., 2022).

Taken together, these results suggest that the parallel modules of dLGN–V1, pLPN–vHVA, and aLPN–dHVA may work to decorrelate spontaneous activity among V1, vHVA, and dHVAs (Figure 2B).

## Hypothesis on developmental mechanisms of parallel thalamocortical connections

How does the LPN–HVA pathway form in parallel with dLGN–V1? This formation may be explained by molecular mechanisms, mainly axon guidance molecules, because LPN neurons need to extend their axons over long distances to reach HVAs. In addition, although it is unknown exactly when projections from the LPN to HVAs are formed, the strong activity propagation from LPN to HVAs at P5 suggests that this pathway may begin to form a few days earlier than P5, similar to the dLGN–V1 projections. To date, no studies have examined Eph/ephrin expression in the LPN during development; however, *in situ* hybridization section images of the thalamic regions containing the dLGN and LPN show clear expression of Eph/ephrin in the LPN (Cang et al., 2005a) and in the cortical regions surrounding V1 (Torii et al., 2009; Dye et al., 2012). These studies suggest a possibility that Eph/ephrin is involved in the formation of LPN–HVA projections. However, the inverse gradient of Eph/ephrin in V1 and individual HVAs is not clear (Torii et al., 2009; Dye et al., 2012), and it is difficult to determine areal boundaries between HVAs based on their expression patterns. Therefore, we speculate that Eph/ephrin may be insufficient to induce LPN projections to all HVAs and that LPN–HVA projections may also be regulated by other molecules. RNA sequencing analysis of dLGN and LPN gene expression at P3 showed that the gene expression patterns were very different (Frangeul et al., 2016). In both newborn and adult mice, the gene expression pattern in the dLGN is similar to that in the lower-order thalamic nuclei of other sensory modalities, whereas the LPN shares a similar gene expression pattern with higher-order thalamic nuclei of other sensory modalities (Frangeul et al., 2016; Phillips et al., 2019). These results suggest that lower- and higher-order thalamocortical pathways are formed by different molecular controls.

Not all thalamocortical pathways are formed by molecular controls alone; retinal activity that propagates from the retina to the dLGN and LPN may be important. Analysis of gene expression patterns in the thalamic nucleus at P3 by binocular enucleation at birth revealed changes in gene expression in both the dLGN and LPN (Frangeul et al., 2016). Furthermore, the gene expression patterns of Eph/ephrin and other genes in the cortex are also altered by binocular enucleation at birth (Dye et al., 2012). These studies indicate that retinal activity affects the expression patterns of axon guidance molecules, suggesting that an interaction between spontaneous retinal activity and molecular expression controls the formation of thalamocortical pathways, dLGN–V1 and LPN–HVA. To elucidate these possibilities, we must comprehensively analyze the gene expression patterns of the dLGN and LPN from prenatal to postnatal periods with and without manipulation of retinal activity, and further identify the effects of loss of function of candidate molecules on the formation of thalamocortical projections.

## Development of the primate brain implicated by mouse studies

Anatomical studies of monkey and human visual pathways using retrograde tracing have demonstrated that feedforward projections from lower to higher cortical areas emerge prenatally during the third trimester of gestation (Burkhalter et al., 1993; Barone et al., 1996). The macaque monkey was born after 165 days of gestation and projections from V2 to V4 were observed at E112. These projections increase slightly with gestational stage and do not involve large-scale axon refinement (Barone et al., 1996; BatardieÌre et al., 2002); this process is largely complete at birth.

However, research focusing on the formation of the primate visual network has been limited because it requires observation during the embryonic period and genetic methods are not as versatile as those for mice. The mouse visual system has a structure similar to that of primates. In both the primate and rodent visual systems, visual inputs to the retina pass through the dLGN to V1. Visual information is then processed by ventral and dorsal pathways, which are formed by numerous HVAs. HVAs in primates have reciprocal connections with the pulvinar, which corresponds to the LPN in mice. Importantly, the primate pulvinar is divided into at least seven subregions and the HVAs of the dorsal and ventral pathways receive projections from different subregions of the pulvinar. Multiple subdivisions of the pulvinar are thought to be proportional to the number of cortical areas in primates.

Does the modular developmental strategy observed in mice provide any clues for understanding the development of the primate visual system which has a more complex structure than that of the mouse visual system? There is a pathway during development from the retina to the middle temporal cortex (MT), also known as the fifth visual area, *via* the pulvinar in the marmoset brain (Bourne and Morrone, 2017). This pathway is strong during development, and diminishes with growth. In addition, the MT is myelinated earlier than other visual areas (Bourne and Rosa, 2006), and the axon guidance molecule Eph/ephrin is strongly expressed in the MT at early developmental stages (Bourne, 2010). This suggests that the retina–pulvinar–MT pathway may correspond to the parallel module observed in mice. Furthermore, functional connectivity analysis of spontaneous activity using functional magnetic resonance imaging in macaque monkeys soon after birth showed that the ventral and dorsal pathway structures were already separated (Arcaro and Livingstone, 2017), which may be due to immature corticocortical connections and spontaneous activity propagation from the pulvinar to each HVA. At present, MT is the only area known to receive early projection from the pulvinar in the neonatal marmosets, and it remains unknown whether other areas receive projections from their respective pulvinar areas already in neonates. Furthermore, the development of corticocortical connections in the primate visual cortex remains unknown. If other areas also receive projections from the pulvinar prior to intercortical connections, this suggests that the modular strategy is also important for forming a complex mammalian brain.

Thus, not only do the visual pathways of mature mice and monkeys have similar structures, they may also correspond to its formation process. The findings from mouse developmental studies may be useful for understanding the development of neural pathways in the primate brain.

## Conclusion

In this review, we summarized the development of neural networks in the mouse visual system. The mouse brain is well-suited for developmental studies, and the formation of visual pathway from the retina to V1 has been investigated in detail. Recently, the developmental process of the visual network involving multiple HVAs and LPN was demonstrated (Murakami et al., 2022). Here, we also discussed the possible roles of higher-order thalamocortical projections in the formation of cortical dorsal and ventral pathways that process different visual features in parallel. Research on the developmental mechanisms (molecular or activity-dependent controls) of corticocortical connections has just begun, and there are many issues that require further clarification to fully understand the mechanisms of visual network development.

## Author contributions

TM and KO wrote the review. Both authors contributed to the article and approved the submitted version.

## Footnotes

1
https://mouse.brain-map.org/
